# Diagnostic relevance of high field MRI in clinical neuroradiology: the advantages and challenges of driving a sports car

**DOI:** 10.1007/s00330-012-2552-9

**Published:** 2012-07-21

**Authors:** Mike P. Wattjes, Frederik Barkhof

**Affiliations:** Department of Radiology, VU University Medical Center, De Boelelaan 1117, 1081 HV Amsterdam, The Netherlands

**Keywords:** Magnetic resonance imaging, Sensitivity and specificity, Brain, Neuroimaging, Field strength

## Abstract

****Abstract**:**

High field MRI operating at 3 T is increasingly being used in the field of neuroradiology on the grounds that higher magnetic field strength should theoretically lead to a higher diagnostic accuracy in the diagnosis of several disease entities. This Editorial discusses the exhaustive review by Wardlaw and colleagues of research comparing 3 T MRI with 1.5 T MRI in the field of neuroradiology. Interestingly, the authors found no convincing evidence of improved image quality, diagnostic accuracy, or reduced total examination times using 3 T MRI instead of 1.5 T MRI. These findings are highly relevant since a new generation of high field MRI systems operating at 7 T has recently been introduced.

**Key Points:**

• *Higher magnetic field strengths do not necessarily lead to a better diagnostic accuracy*.

• *Disadvantages of high field MR systems have to be considered in clinical practice*.

• *Higher field strengths are needed for functional imaging, spectroscopy, etc*.

• *Disappointingly there are few direct comparisons of 1.5 and 3 T MRI*.

• *Whether the next high field MR generation (7 T) will improve diagnostic accuracy has to be investigated*.

Since approval by the U.S. Food and Drug Administration (FDA) more than a decade ago, the installation and use of high field MR systems operating at 3 Tesla (T) for research purposes and more recently in clinical practice have dramatically increased. Based on the theoretical substantial increase in signal-to-noise ratio (SNR) compared to standard magnetic field strengths (1.5 T and below), the most relevant sales pitch by vendors was the achievement of better image quality and the ability to reduce MR data acquisition and overall examination times when using 3 T. Extensive research reflected in a tremendous number of original articles has been performed and collectively demonstrates the benefits but also challenges of 3 T MRI. Particularly in the field of neuroradiology, the benefits of 3 T imaging seem quite obvious, not only to obtain better quality routine structural imaging (e.g. of inflammatory lesions), but especially for applications with inherently low SNR, such as susceptibility weighted imaging (SWI), MR angiography (MRA), diffusion tensor imaging (DTI), functional MRI (fMRI), and MR spectroscopy [1–5]. However, still up for debate is the crucial question of the diagnostic relevance of 3 T MRI or in other words “Does 3 T MRI lead to a more sensitive (earlier) and/or more specific diagnosis of certain diseases?”.

In the current issue of *European Radiolog*y, Wardlaw and colleagues present an exhaustive review of research comparing 3 T MRI with 1.5 T MRI in the field of neuroradiology [6]. The authors evaluated 150 articles assessing a total of 4,500 subjects published between January 2000 and October 2010 to find objective evidence that MRI at 3 T provides higher diagnostic accuracy when compared to 1.5 T. Interestingly, they found no convincing evidence of improved diagnostic accuracy or reduced total examination times, both of which constitute the most commonly used arguments put forward by 3 T manufacturers. In fact, data directly comparing both field strengths were scarce, and most studies employed different types and/or generations of technologies (e.g. coils), different pulse sequences, and differences in contrast agents. Most studies were small, had no randomisation or blinding, and case selection (or exclusion bias) was not reported; beyond that, diagnostic impact and cost-effectiveness were typically not evaluated.

Although these results may seem surprising at first glance, they may not be completely unexpected. A direct comparison of different magnetic field strengths in terms of diagnostic accuracy is almost impossible. The physical changes occurring when moving to higher magnetic field strength (e.g. tissue relaxation times, specific absorption rate) make it crucial to adjust the pulse sequences and MR protocol in terms of repetition and times, but also require a choice between spatial and temporal resolution to achieve “comparable” conditions [1, 7]. However, from a formal point of view, it is arguable whether these conditions are really comparable in terms of a side-by-side 1.5 versus 3 T comparison. In addition, it is important to realise that there are other important factors that substantially contribute to image quality such as coil technology, field homogeneity, and multi-transmission technology [8]. Differences in image quality and MR performance can even be observed among MR systems of different vendors operating at the same field strength [9].

Regardless of the fact that a straightforward side-by-side comparison of 1.5 T and 3 T is very difficult from a methodological point of view, we should ask ourselves whether it is necessary and useful to formally test all possible differences in terms of diagnostic accuracy between both field strengths. Advantages and disadvantages of higher magnetic field strengths are well known and incontestable. The higher SNR at 3 T allows imaging at a higher spatial resolution and the application of parallel imaging techniques as well as routine use of 3D sequences [10]. It has been conclusively shown that 3 T shows more (small) inflammatory white and grey matter lesions and offers a better visualisation of smaller arterial branches on time-of-flight and contrast-enhanced MR angiography [11–14]. Despite the fact that this might not lead to an earlier diagnosis of MS or an earlier detection of aneurysm or vessel malformation with significant therapeutic consequence, (neuro)radiologists would opt for the better image quality if this came at the same cost and level of safety, since it makes the reading procedure easier and more reliable. This is true even for standard clinical examinations but becomes even more obvious for advanced and quantitative MR methods substantially benefiting from improved SNR, such as MR spectroscopy, perfusion MR (including ASL), and fMRI. Disappointingly, studies dealing with these methods were almost exclusively performed in the research setting, and in particular, comparative studies in the clinical setting using these techniques are lacking.

Simply speaking, the situation is (to a certain extent) comparable to one’s choice of car. The advantages of a sports car with a lot of horsepower are quite evident. Sport cars are relatively expensive and need more fuel. However, they are also faster than other cars and allow you to reach your destination earlier under pleasant conditions. On the other hand, in a traffic jam or with bad road infrastructure, the advantages of a sports car will disappear and the driver will have to deal with substantial challenges. In those circumstances, it might be better to drive an economical car or use public transportation. The described bad street conditions for a sports car correspond to certain 3 T applications such as spinal cord imaging. Negative bias among the studies examined includes the absence of 3 T spinal cord data. The situation is comparable to certain body applications such as abdomen and pelvis imaging [15]. For those reasons, a small hospital that can afford only a single MR system should not consider purchasing a 3 T system since it will not be able cover all clinical demands.

We have to be aware that the high standard of clinical trial methodology, which is rightly required for treatment trials proving the superiority of one drug compared to placebo or another drug, is not simply transferable to diagnostic imaging comparing one method with another. Technological improvements are occurring continuously, and it is impossible to systematically compare each new technique with an existing one, certainly if the advantages are obvious and come without additional costs or penalties. Only major steps require validation, and the discussion of the diagnostic benefit of higher magnetic field strength will shift gears when whole-body ultra-high field MR systems operating at 7 T need to be compared with 3 T [16, 17]. Once again, certain advantages of 7 T are quite obvious and promising even in the clinical setting. However, the 3 T sports car mutates into a 7 T Formula One racing car, which makes daily use even more challenging. The major question is *under what circumstances* are the increased costs of 7 T and the inherent limitations in coverage and safety outweighed by diagnostic benefits.
